# Adding Some “Splice” to Stress Eating: Autophagy, ESCRT and Alternative Splicing Orchestrate the Cellular Stress Response

**DOI:** 10.3390/genes12081196

**Published:** 2021-07-31

**Authors:** Elias Habib, Allyson Cook, Sabateeshan Mathavarajah, Graham Dellaire

**Affiliations:** 1 Department of Pathology, Dalhousie University, Halifax, NS B3H 4R2, Canada; ehabib@dal.ca (E.H.); a.cook@dal.ca (A.C.); smathavarajah@dal.ca (S.M.); 2 Department of Biochemistry and Molecular Biology, Dalhousie University, Halifax, NS B3H 4R2, Canada

**Keywords:** autophagy, ESCRT, alternative splicing, cancer, neurodegenerative and eye disease

## Abstract

Autophagy is a widely studied self-renewal pathway that is essential for degrading damaged cellular organelles or recycling biomolecules to maintain cellular homeostasis, particularly under cellular stress. This pathway initiates with formation of an autophagosome, which is a double-membrane structure that envelopes cytosolic components and fuses with a lysosome to facilitate degradation of the contents. The endosomal sorting complexes required for transport (ESCRT) proteins play an integral role in controlling autophagosome fusion events and disruption to this machinery leads to autophagosome accumulation. Given the central role of autophagy in maintaining cellular health, it is unsurprising that dysfunction of this process is associated with many human maladies including cancer and neurodegenerative diseases. The cell can also rapidly respond to cellular stress through alternative pre-mRNA splicing that enables adaptive changes to the cell’s proteome in response to stress. Thus, alternative pre-mRNA splicing of genes that are involved in autophagy adds another layer of complexity to the cell’s stress response. Consequently, the dysregulation of alternative splicing of genes associated with autophagy and ESCRT may also precipitate disease states by either reducing the ability of the cell to respond to stress or triggering a maladaptive response that is pathogenic. In this review, we summarize the diverse roles of the ESCRT machinery and alternative splicing in regulating autophagy and how their dysfunction can have implications for human disease.

## 1. Introduction

### 1.1. Overview of Autophagy

Autophagy is categorized into three distinct degradative systems referred to as micro-, macro-, and chaperone-mediated autophagy, which act to recycle and degrade damaged organelles and biomolecules to maintain cellular homeostasis. Macroautophagy involves the formation of a double-membrane autophagosome, while microautophagy and chaperone-mediated autophagy are selective and operate via the direct engulfment of the lysosomal membrane and the chaperone-dependent selection of cytosolic components, respectively [1]. All three subtypes of autophagy are activated by cellular stresses that perturb normal physiological processes and these include nutrient deprivation, hypoxia, mitochondrial damage, heat stress and intracellular pathogens [2,3]. Autophagy is an evolutionarily conserved process, first observed in *Saccharomyces cerevsiae*, *Pichia pastoris* and *Hanensula polymorpha* [4]. Others have since shown using genetic, morphological and comparative genomic data that autophagy machinery exists throughout eukaryote life, including plants, amoebozoan, fungi and metazoan [4]. Consistent with autophagy’s central role in maintaining cellular homeostasis, aberrant autophagy can play a role in cancer progression and the development of neurodegenerative diseases such as in Parkinson’s disease [5,6]. In addition, the endosomal sorting complexes required for transport (ESCRT) machinery has also been shown to play a prominent role in the regulation of autophagy and other cellular processes such as cell division that involve the fusion of cell membranes [7], which we discuss in detail later in this review.

**Figure 1:**
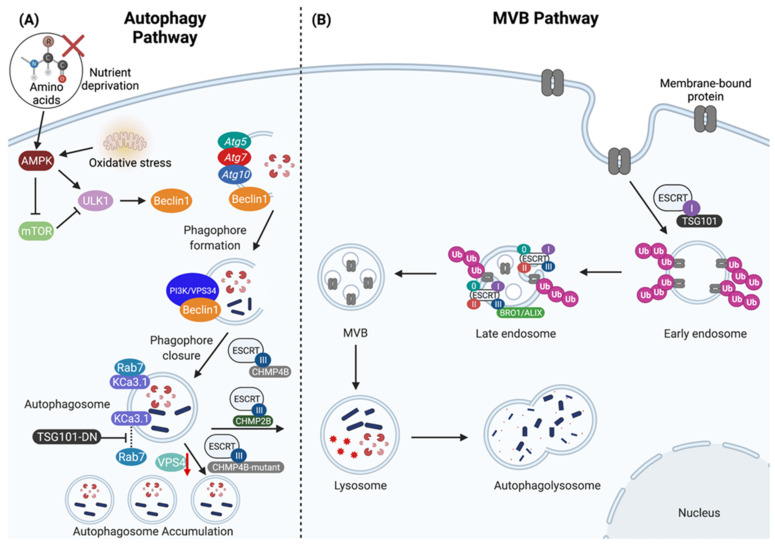
The regulation of lysosomal-mediated degradation through the autophagy and multivesicular body (MVB) pathways. (**A**) The autophagy pathway is induced by AMP-activated protein kinase (AMPK) and inhibited by mammalian target of rapamycin (mTOR). Nutrient deprivation and oxidative stress promote AMPK activity, in turn activating the UNC-51 like kinase 1 (ULK1) complex, which then recruits *Atg5*, *Atg7*, *Atg10* and phosphorylates Beclin1 to promote further recruitment of the PI3K/VPS34 complex to enable phagophore formation. CHMP4B is a core component of ESCRT-III that further assists in mediating phagophore closure. The fusion between the autophagosome with the lysosome is mediated by CHMP2B to form the autophagolysosome, which contains cytosolic content destined for degradation. The absence of functional CHMP4B (CHMP4B-mutant) and either knock-down of VPS4 expression (red down arrow) or expression of dominant negative VPS4 or TSG101 (TSG101-DN) prevents autophagosome-lysosome fusion resulting in autophagosome accumulation, and failure to degrade cargo such as the calcium-activated potassium channel 3.1 (KCa3.1). (**B**) The biogenesis of MVBs and sorting of ubiquitinated cargo is regulated through the ESCRT pathway. The ESCRT machinery are grouped into four categories: ESCRT-0, ESCRT-I, ESCRT-II, and ESCRT-III. TSG101 is a member of the ESCRT-I complex that facilitates the sorting of ubiquitinated membrane-bound proteins into early endosomes. The ESCRT machinery sequentially assembles on the late endosome where it mediates the biogenesis of MVB vesicles and the sorting of ubiquitinated membrane-bound proteins into intralumenal vesicles (ILVs), and the ESCRT-III complex is involved in the recruitment of BRO1/ALIX to assist in the biogenesis of ILVs. After the biogenesis of MVBs and the sorting of ubiquitinated proteins into ILVs, MVBs are then routed into the lysosome for degradation. Created with BioRender.com.

As mentioned above, autophagy is an intracellular degradation system that transports cytosolic contents to the lysosome [1,8,9]. The stress-induced autophagy pathway is initiated through the activation of the AMP-activated protein kinase (AMPK) and inhibition of mammalian target of rapamycin (mTOR) (described in detail below) in response to a multitude of cellular stresses, such as nutrient deprivation, heat shock, mitochondrial damage [2,3,10]. These early signaling events trigger the formation of the phagophore (i.e., isolation membrane), the structure that proceeds the autophagosome (Figure 1A). The phagophore elongates to surround the cytosolic contents that have been selected for degradation. Formation of the phagophore requires not only a signaling event but both the serine/threonine protein kinase UNC-51 like kinase 1 (ULK1) complex and four autophagy related genes, *Atg5*, *Atg7*, *Atg10* and *Atg12*, commonly known as a “ubiquitin-like conjugation system” [9,10,11,12,13,14]. The ULK1 complex recruits the PI3K/VPS34 complex and translocate to autophagy initiation sites upon induction [13]. In combination, these complexes result in the formation of the phagophore.

Phagophore formation may begin via the pre-autophagosomal structure (PAS), a structure that has been well-characterized in yeast [1]. The mammalian phagophore originates from an isolation membrane that acquires lipids from the endoplasmic reticulum exit sites, mitochondria, ER-mitochondria contact sites, ER-Golgi intermediate, Golgi apparatus, and plasma membrane [15]. Regardless of its place of origin, the phagophore is a double membraned structure that develops through a large macromolecular complex containing the class III phosphoinositide 3-kinase (PI3K) vacuolar protein sorting 34 (*Vps34*) [8,9]. The complete development of the phagophore is a result of the recruitment and function of light chain 3 (LC3), which is responsible for membrane tethering and hemifusion. The transition from the phagophore to the autophagosome is dependent on these processes, thus leading to the full sequestration of the cytosolic contents that have been selected for degradation [1,9]. The autophagosome then fuses with a lysosome to create the autophagolysosome, such that the concentration of acidic hydrolytic enzymes is high and rapid degradation of autophagic cargo can occur [1] (Figure 1A).

As briefly discussed above, AMPK and mTOR are central regulators of autophagy in the context of cellular stresses including oxidative stress (e.g., due to mitochondrial dysfunction) and nutrient deprivation (e.g., absence of amino acids) in yeast and mammalian organisms [1,8,9,16,17]. AMPK senses nutrient or oxidative stress and then in turn activates ULK1 by direct phosphorylation [10] and indirectly by inhibiting mTOR activity [18]. AMPK inhibition of mTOR activity is both direct by phosphorylation of regulator associated protein of mTOR (Raptor) and indirectly by phosphorylation of tuberous sclerosis complex 2 (TSC2) [10,19,20,21]. The regulation of autophagy via mTOR is mediated through two complexes, mTOR complex 1 (mTORC1) and mTOR complex 2 (mTORC2) [9]. Raptor is a binding partner to mTORC1 and is required for mTOR signaling and its phosphorylation by AMPK directly inhibits mTORC1 [19,21,22]. TCS2 is a tumor suppressor protein that dimerizes with TSC1, and genes encoding both proteins are found mutated in forms of tuberous sclerosis [23,24]. AMPK phosphorylation of TSC1 activates the TSC1-TSC2 complex, in turn inhibiting mTORC1 through its Rheb-GAP activity [20,25]. However, TSC1-TSC2 can also activate mTORC2 independently of this activity [26]. As such, the TSC1-TSC2 complex integrates signals from various pathways, including AKT and AMPK to monitor incoming growth factor signals and relay that information so mTORC1 is properly controlled [27]. Although extensive research has firmly established a central role for mTORC1 in the negative regulation of autophagy via inhibitory phosphorylation of ULK1, much less is known regarding the role of mTORC2 in this pathway [10]. 

In one study, it was shown that activation of mTORC2 results in the indirect inhibition of autophagy by activating AKT that in turn suppresses FOXO3-mediated transcription of autophagy genes [28]. Yet in a more recent study, mTORC2 was shown to regulate mitochondrial homeostasis via activation of serum- and glucocorticoid-inducible kinase 1 (SGK-1), and inactivation of mTORC2 induced autophagy through mitochondrial dysfunction and oxidative stress [29]. mTOR-independent regulation of autophagy can also occur, and has been shown to be dependent upon inositol signaling, changes to intracellular calcium, and elevation of cyclic AMP [9]. The focus of this review will be on mTORC1-dependent signaling in autophagy.

### 1.2. Overview of ESCRT

The ESCRT pathway is also highly conserved from yeast to humans and is essential for a number of diverse cellular processes including multivesicular body (MVB) biogenesis and sorting of ubiquitinated membrane proteins into intralumenal vesicles (ILVs) (Figure 1B), viral budding, cytokinesis, transcription and cell cycle control [7,11,30,31]. The ESCRT pathway is grouped into four categories: ESCRT-0, which regulates MVB biogenesis; ESCRT-I, which is also responsible for MVB biogenesis and for sorting ubiquitinated cargo into MVBs; ESCRT-II, which is responsible for assisting ESCRT-I in MVB biogenesis; and finally, ESCRT-III, which functions in cellular abscission and contributes to viral budding when cells are infected [30,31,32,33,34]. Furthermore, the ESCRT machinery is also required for autophagosome-lysosome fusion and the consequent degradation of sequestered cytosolic contents [7]. However, the dysregulation of ESCRT leads to the accumulation of damaged cellular organelles, such as mitochondria [7]. Inactivation of ESCRT machinery results in metabolic stress, a mechanism by which autophagy becomes activated [11]. 

The formation of the autophagosome requires the induction, trafficking and fusion of membrane-bound organelles. One set of machinery for membrane-bound organelle trafficking involves the ESCRT pathway, which is responsible for protein sorting, deformation of membranes and their scission [30,35,36,37]. It has been previously shown that when ESCRT machinery is disrupted in mammals, there is an accumulation of autophagosomes, indicating a significant role of ESCRT in the regulation of autophagy [11,38,39,40]. A product of disrupted autophagy is dysfunctional mitochondria, and the accumulation of misfolded and aggregated proteins, which increase in number during aging and are hallmarks of neurodegenerative diseases, such as Alzheimer’s and Parkinson’s disease [41,42]. Neurons in the central nervous system (CNS) of patients with these neurodegenerative diseases often contain both damaged mitochondria and aggregates of amyloid proteins, which is likely a result of impaired autophagy.

Disruption of autophagy through the depletion of ESCRT-III complex proteins has been directly linked to the neurodegeneration in animal models and in human mutations of the charged *multivesicular body protein 2B gene* (*CHMP2B*) (Table 1). CHMP2B encodes an ESCRT-III protein, and its mutation has been associated with frontotemporal dementia linked to chromosome 3 (FTD3) and amyotrophic lateral sclerosis (ALS) [43,44,45,46]. Recent advances in our understanding of CHMP2B have helped connect ESCRT-mediated autophagy (and mitophagy) to neurodegeneration. An indicator of impaired autophagy is the accumulation of autophagosomes, or damaged organelles such as mitochondria. Rusten and Stenmark proposed four fundamental models to explain the mechanisms behind why the inactivation of ESCRT machinery causes the accumulation of autophagosomes. These models include: an induction model, phagophore closure, autophagosome fusion and a lysosome biogenesis model [11]. There are several possibilities when considering the link between autophagy and the ESCRT machinery; however, in this review we will specifically discuss how ESCRT-I and III promotes autophagosome fusion.

In order for biomolecules and organelles in the cytoplasm to be recycled or degraded, they need to be engulfed within an autophagosome, which in turn must fuse with the lysosome. Consequently, inhibition of lysosomal fusion results in the accumulation of autophagosomes containing un-degraded molecular cargo within the cell [11]. Soluble N-ethylmaleimide sensitive factor attachment protein receptor (SNARE) complexes are currently thought to mediate autophagosome fusion [11]. However, prior to SNARE formation, the link between the autophagosome and endolysosome is thought to be controlled by Rab7 (a marker of the late endosome) through a plasma membrane associated calcium-activated potassium channel 3.1 (KCa3.1, also known as KCNN4) [54] (Figure 1B). Balut et al. demonstrated that KCa3.1 is targeted to the lysosomes for degradation in human embryonic kidney cells (HEK) and human dermal microvascular endothelial cells (HMEC-1) [54]. From this, extensive research was conducted to demonstrate that there is a close association between Rab7 and KCa3.1, and that the overexpression of a dominant negative ATPase defective Vps4 (an ESCRT disassembly protein) substantially decreased the rate at which KCa3.1-positive membranes were degraded, likely due to the failure of autophagosomes-lysosome fusion. This is consistent with previous findings that expression of dominant negative Vps4 in flies could induce autophagosome accumulation [40]. Furthermore, expression of a dominant negative C-terminal fragment of the tumor susceptibility gene 101 (TGS101), a protein involved in the ESCRT-I pathway (discussed below) (Figure 1A), inhibited KCa3.1 degradation [54] Thus, these studies demonstrate that ESCRT proteins play a critical role in autophagosome-lysosomal fusion events and consequently the degradation of membrane proteins like KCa3.1.

The ESCRT-III pathway is also implicated in autophagy during phagophore closure and the lysosomal targeting of KCa3.1 [54,55]. For example, overexpression of a dominant negative cyan fluorescent protein fusion of the ESCRT-III protein CHMP4B was shown to induce enlarged endosome structures and inhibited the lysosomal target of the KCa3.1 channel [54]. In the context of mitophagy, it was also shown that depletion of ESCRT-III protein CHMP2A lead to increased association of CHMP4B with mitophagosomes and resulted in impaired phagophore closure around damaged mitochondria [55] (Figure 1A).

### 1.3. Overview of Alternative Splicing

Similar to autophagy, alternative splicing of mRNAs can be triggered in response to endogenous or exogenous cellular stress [56,57,58]. Alternative splicing is a process that results in several protein isoforms being expressed by the same gene [59,60]. Precursor mRNAs (pre-mRNAs) encoded by genes in eukaryotic genomes are spliced to form mature mRNAs [61]; a process carried out by the spliceosome, a ribonucleoprotein complex that removes introns (noncoding sequences) and joins exons together (coding sequences) [56,60,61]. Alternative splicing allows for an added layer of gene regulation, as the cell can generate multiple transcripts that encode protein variants from the same source of genomic DNA. In addition to coding sequences, these alternative transcripts can also encode different 5′ and 3′ untranslated regions (UTRs) that may affect transcript stability or translation. This tightly regulated process was recently linked to mRNA transcripts encoding isoforms of genes that control autophagy such as Beclin1 [62], which in turn may influence the autophagy pathway or have entirely independent functions within the cell. Thus, these findings indicate that alternative splicing represents a novel regulatory mechanism for altering protein isoform expression, and potential function, of the autophagy machinery.

## 2. Alternative Splicing and Autophagy

### 2.1. Genes Associated with Autophagy Are Alternatively Spliced

The activation of autophagy is initiated by the protein kinase AMPK in response to the cellular stresses as mentioned above. In addition, the cellular response to these same stresses also involves changes to the transcriptional landscape [63,64]. These transcriptional changes have been associated with altered mRNA splicing, which results in alternative splicing of genes that regulate autophagosome formation. For instance, the yeast *Atg6* ortholog, *Beclin1* can be alternatively spliced and functions primarily as a regulator of autophagy [65] (Table 2). Alternative pre-mRNA splicing of the *BCN1* gene encoding Beclin1 is responsible for converting Beclin1 from an inducer of autophagy to a regulator of mitophagy (degradation of mitochondria via selective autophagy) [66]. Furthermore, Beclin1 isoforms have differential interactions with class III PI3K in the context of starvation induced autophagy [62].

Along with Beclin1, splice variants of *the autophagy-related gene 8* (*Atg8*) family gene member *MAP1LC3B* (also known as LC3B) can also alter autophagic activity (reviewed by Paronetto et al. [66]). *LC3B* is required for autophagosome biogenesis; however, its exact function remains unknown [67]. As a result of the current data on the function of *LC3B*, it is speculated that it is required for phagophore formation, therefore, this limiting effect on autophagy may lead to incomplete closure of the phagophore [67]. In a third and fourth example, the genes encoding *ATG5* and *ATG7* are proteins required for autophagy (Table 2; Figure 1A) have been shown to be alternatively spliced with consequences for autophagy [66]. For example, ATG5-deficient prostate cancer cell line DU145 expresses two different *ATG5* alternative transcripts (missing exons 3, or both exons 3 and 6) and exhibits impaired autophagy initiation in response to valproic acid; a defect that could be complemented with the canonical full length *ATG5* mRNA [69]. Additionally, an autophagy defect was found to be associated with alternative splicing of *ATG7* as a result of mutations in the splicing factor U2AF35; an event that occurs in several cancers [68]. From these data, it is tempting to speculate that alternative splicing may play a broader role in modifying other autophagy inducing genes, or genes responsible for autophagosome fusion, including those in the ESCRT pathway. Given the rapid ability of alternative pre-mRNA splicing has on modifying protein function in response to cellular stress-including nutrient starvation, heat shock, oxidative and osmotic stress-further exploration on this topic is required to gain a deeper understanding of the relationship between the autophagic pathway and alternative splicing [57,58,70,71,72].

### 2.2. ESCRT-III Alternative Splicing Inhibits Autophagy-mediated Cell Death in Yeast

CHMP3/Vps24 is an ESCRT-III protein that interacts with CHMP2A and promotes CHMP2A binding to membranes [73]. CHMP2A and CHMP3 form helical polymers that contribute the ability of ESCRT-III proteins to constrict membranes to facilitate membrane fission events in the cell [74]. In 2007, Khoury et al. reported that one of the genes identified in a Bax suppressor screen was a novel alternatively spliced isoform of *CHMP3* [75]. The alternatively spliced transcript encodes a 156 amino acid variant of CHMP3/VPS24 (referred to as VPS24β) that is truncated and lacking the N-terminal lipid binding domain. In a yeast model, the group ectopically expressed both the truncated and canonical full-length human CHMP3/VPS24 transcripts; and found that while the truncated VPS24β isoform could not compensate for the functions of yeast Vps24, full-length human CHMP3/VPS24 did complement autophagy defects. Thus, the alternatively spliced human VPS24β isoform of CHMP3 appears to play a novel ESCRT-III-independent role in the cell through regulation of Bax. Human Bax is known to have pleiotropic effects in yeast when it is heterologously expressed, which is in part due to the induction of autophagy [76,77]. Similarly, Bcl-2 is also known to play a role in both autophagic and apoptotic cell death [78]. In the presence of the human *CHMP3* isoform VPS24β but not the full-length *CHMP3* transcript, the growth inhibitory effects of Bax were suppressed.

Although the mechanistic details of how VPS24β suppresses Bax activity remains unknown, it may involve dysregulated autophagy. For example, when CHMP3/VPS24 was depleted in HeLa cells, they exhibited impaired autophagic flux and accumulate aggregates of the TDP-43 protein marked by the autophagy receptor p62 and ubiquitin [38] (Table 1). These data also have relevance for both frontotemporal lobar degeneration (FTLD-U) and ALS, as neurons from patients with these diseases accumulate ubiquitin- and/or p62- positive neuronal cytoplasmic inclusions [79]. TDP-43 was also identified as one of the major proteins accumulating in FTLD-U and ALS [80]. This connection between ESCRT-III protein isoforms, protein aggregates and alternative splicing in FLTD-U and ALS will be expanded upon in more detail below in the context of splicing, autophagy and human disease.

## 3. Alternative Splicing Regulates ESCRT Recruitment to NPC Assembly Sites

Nuclear pore complexes (NPCs) are required for the translocation of macromolecules in and out of the nucleus [81]. NPCs are large channels spanning the nuclear envelope and are comprised of numerous copies of ~30 proteins named nucleoporins. The disruption of NPC structure is linked to aging and several diseases [81,82]. Nuclear envelope integrity relies on membrane remodeling by the ESCRTs, that survey and seal nuclear envelope holes–ultimately contributing to the maintenance of nuclear pore complexes and recently, the ESCRT protein CHMP7 has been linked to NPC turnover [83].

**Figure 2:**
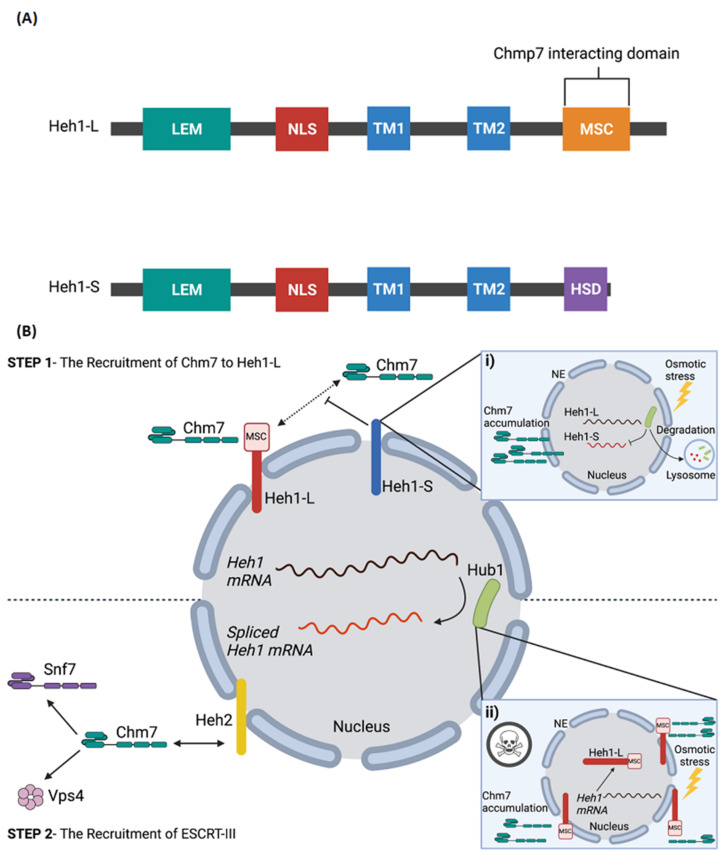
Alternative splicing of Heh1 regulates Chm7 recruitment to NPC assembly sites. (**A**) The domain architecture of Heh1-L and Heh1-S [87]. Alternative splicing of *Heh1* results in two variants: a long form (Heh1-L) and a short form (Heh1-S). The splicing events causes a shift in the open reading frame of Heh1-S that results in the deletion TM2 and the C-terminal MSC domain (Chm7 interacting domain), and the addition of the Heh1-S specific domain (HSD). (**B**) Heh1-L recruits Chm7 through its MSC domain (Step 1); after which Chm7 directly interacts with Heh2 to recruit downstream factors that promote ESCRT-III assembly, including Snf2 and Vps4, to facilitate NPC turnover (Step 2). In contrast, Heh1-S inhibits the recruitment of Chm7 as a result of the missing C-terminal MSC domain (i). This balance of Heh1 splice isoforms is regulated during heavy metal and oxidative cell stress to regulate the accumulation and retention of Chm7 at the nuclear envelope. However, under osmotic stress, Hub1 is exported out of the nucleus and degraded, an event that inhibits alternatively splicing of Heh1 and resulting in only Heh1-L being expressed. In the absence of Heh1-S and expression of Heh1-L alone, Chmp7 is excessively recruited to the nuclear envelope which is toxic to the cell (ii). Created with BioRender.com.

The yeast homolog of human CHMP7 is Chm7, which is recruited to sites of NPC assembly by the integral inner nuclear membrane proteins Heh1 and Heh2 [83]. Here, Chm7 contributed to the sealing of defective NPCs to maintain cell viability. Chm7 is excluded from the nucleus through active CRM1-mediated export [84]. In the cytosol, it normally interacts with Chm7 through a winged-helix MSC domain (for MAN1-Src1 C-terminal) found in its C-terminus (Figure 2A). When exposed to the inner nuclear membrane, Heh1 locally activates Chm7. This interaction is partly facilitated by the phosphatidic acid binding activity of Chm7 to the inner nuclear membrane [85]. After recruitment is established, Chm7 directly interacts with Heh2 to then recruit downstream factors that promote ESCRT-III assembly such as Snf7 and Vps4 [83] (Figure 2B). As such, this ESCRT-III quality control surveillance system maintains the integrity of the nuclear envelope in the event of membrane holes and misassembled NPCs.

NPC recycling is also regulated through alternative splicing. Specifically, an unconventional ubiquitin-like protein known as Hub1 orchestrates a splicing event in the transcript of the Heh1 protein that in turn controls yeast Chm7 recruitment to the NPC [85,86]. Two splice isoforms for *Heh1* are found in yeast, which encode a long (Heh1-L) and a short (Heh1-S) form of the Heh1 protein [87]. Hub1-mediated alternative splicing is responsible for the Heh1-S isoform, which has a unique 49 amino acid insertion not found in Heh1-L and lacks the C-terminal MSC domain required for interaction with Chm7 [87] (Figure 2B). During normal cell growth, in cells where ESCRT-III activity is absent, this alternatively spliced Heh1 transcript dominates and prevents excessive recruitment of Chm7 to the nuclear envelope, which was shown to be toxic to the cell in the absence of the alternatively spliced Heh1-S. Therefore, Heh1 alternative splicing may act as safety switch to maintain tight regulation of Chm7 recruitment to sites of NPC assembly. Hub1-mediated splicing occurs in response to oxidative, and heavy metal stress as well [86]. Thus, it is possible that the upregulation of Hub1 is a general response to extreme stress that limits the toxic prolonged association of Chm7 with NPC sites [86]. However, under osmotic stress, the mammalian homolog of Hub1, called UBL5, is exported out of the nucleus and degraded [88,89]. Thus, these results indicate that under different stress conditions, mechanisms for the maintenance of the nuclear envelope and NPC turnover may differ. Whether or not Hub1/UBL5-mediated control of CHMP7 recruitment to the NPC is conserved in humans is still unknown.

The autophagy pathway also seems to be directly involved in Chm7 function at the nuclear envelope. In the Costanzo et al. genetic interaction map in *Saccharomyces cerevisiae* (synthetic genetic arrays), a negative genetic interaction was identified between *Atg17* and *Chm7* [90]. Atg17 plays an important role in binding Atg1 to phagophore assembly sites, where autophagosome formation is initiated in yeast. However, it was recently discovered to have a secondary role in Snx4-mediated nucleophagy in yeast [91]. Furthermore, new work by Lee et al. indicates that the turnover of defective NPC occurs through selective autophagy that requires ESCRT-III [92]. It is possible that Chm7 is involved in Atg17-mediated nucleophagy, and this is connected to its role in NPC surveillance and turnover. Since nucleophagy is still a poorly understood area of selective autophagy, emerging work that examines the role of Chm7 in the process may help solve some of the mechanistic gaps remaining in the pathway.

## 4. Autophagy, ESCRT, and Alternative Splicing in Human Disease

### 4.1. Aberrant TSG101 Splicing in Cancer

Tumor susceptibility gene 101 (TSG101) is a member of the ESCRT-I complex and mediates a variety of cellular functions, such as promoting cytokinesis, cell cycle progression and proliferation, and eliciting endosomal trafficking and viral budding [93,94]. TSG101 is characterized as a typical E-type Vps protein, which facilitates the transport of membrane proteins into vesicles [95]. Importantly, TGS101 is a member of the ESCRT-I complex, which is involved in the sorting of ubiquitinated proteins to endosomes and the formation of MVBs [93,94]. As mentioned previously, dysregulation of ESCRT proteins have been reported in human diseases, such as cancers and neurodegenerative diseases. TSG101 is an essential protein for the clearance of potentially toxic organelle remnants and protein aggregates [94] and its aberrant splicing has been shown to play a role in cancer progression and tumorigenesis in multiple malignancies [93,96] (Table 1).

Tsg101 is considered to be a tumor suppressor as its functional inactivation has been shown to promote metastatic tumors in mice [47]. However, the deletion of *Tsg101* in mice is embryonic lethal impacting the growth, proliferation, and survival of embryonic cells, and conditional knockout in adult mammary epithelial cells impairs mammogenesis [97]. Rather than being deleted, the TSG101 protein is highly expressed in a range of human cancers, typically in association with aberrantly spliced transcripts, and TSG101 splice variants have been identified in leukemia as well as breast, cervical, lung, ovarian and prostate cancers [48,49,96,98,99,100]. The most highly expressed TSG101 splice variant in human tumor tissues is TSGΔ154-1054, which results in a deletion of nucleotides 154 to 1054 [48,96,97,98]. Elevated TSGΔ154-1054 expression has been observed during the progression of cervical neoplasia, suggesting this TSG101 splice variant may play a direct role the development of cervical cancer [48]. Thus, the aberrant accumulation of TSG101 rather than its loss is linked to cancer in humans.

There are two major routes for post-translational regulation of TSG101 expression: TSG101-associated E3 ligase (Tal)- and MDM2-mediated proteasomal degradation [93]. Tal negatively regulates the TSG101 levels by targeting the protein for ubiquitination. However, Chua et al. [101] reported in 2019 that TSG101 accumulation results from TSGΔ154-1054 by competitively binding to Tal and not MDM2, thus protecting TSG101 from polyubiquitination and proteasomal degradation, which in turn promotes malignancy. In addition, Tal cannot target TSGΔ154-1054 for degradation either, regardless of Tal binding to TSGΔ154-1054 [93]. The reason for this is that the lysine residues in the C-terminal S-box domain of TSG101, that are targeted for Tal-mediated polyubiquitination [102], are absent in TSGΔ154-1054 because of premature translational termination. Therefore, when TSGΔ154-1054 inhibits Tal, both TSGΔ154-1054 and TSG101 accumulate in the cell.

How the accumulation of TSG101 contributes to cancer is likely a function of its role in autophagy-mediated protein degradation. For example, TSG101 recognizes specific ubiquitinated proteins and targets them towards lysosomal degradation, including internalized growth factor receptors. Oh et al. [49] reported that the overexpression of TSG101 could inhibit the downregulation of the activated epidermal growth factor receptor (EGFR), leading to increased phosphorylation of EGFR and subsequent activation of the MAP kinase cascade. In aging mice, the overexpression of TSG101 also increased malignant transformation of the mammary epithelia through aberrant EGFR signaling [49]. In conclusion, the mis-splicing of TSG101 in malignant cells contributes its accumulation that in turn impacts growth factor signaling pathways to promote oncogenesis.

### 4.2. CHMP2B Mis-Splicing Dysregulates Autophagy in FTLD

As previously discussed, ESCRT-III is essential for the formation and abscission of intralumenal vesicles (ILVs) [103]. As a result, ESCRT-III gives rise to MVB that are then routed into the lysosomes for degradation. The ESCRT complexes were originally discovered in yeast as class E Vps proteins. Yeast Vps proteins have one or more human orthologues, indicating a conserved association with the ESCRT pathway in mammals. Importantly, the CHMP2B gene is one of the two human orthologues of the yeast Vps2 protein, which is encoded to form the components of the heteromeric ESCRT-III complex. The CHMP2B protein is highly expressed in major brain regions such as the hippocampus, frontal and temporal lobes, and cerebellum. CHMP2B mutations are also implicated in neurodegenerative disorders, including FTLD, progressive supranuclear palsy (PSP), and corticobasal degeneration (CBD) [50] (Table 1).

FTLD is a group of clinically and neuropathologically heterogeneous disorders, which is characterized by abnormalities in behaviour or language caused by the degeneration of the frontal and anterior temporal lobes [104,105]. After Alzheimer’s disease (AD), FTLD is the second most prevalent cause of dementia in younger people. However, unlike AD, FTLD has a strong genetic basis, such that 40–50% of cases are family cases [105,106]. FTLD is associated with three neuropathological entities: FTLD-tau, FTLD-TDP (also known as FTLD-Ubiquitin), and FTLD-FUS. FTLD-tau results in the intraneuronal accumulation of filamentous, hyperphosphorylated microtubule-associated protein Tau, which assembles into insoluble filaments that accumulate in neurons or glial cells. The phenotypic variability of FTLD-tau among patients has resulted in categorizing FTLD-tau into subtypes. The most common subtypes of FTLD-tau include Pick’s disease (PiD), CBD, and PSP. Patients who develop FTLD-tau meet the diagnostic criteria for the behavioral variant of frontotemporal dementia (bvFTD), which often present behavioral and cognitive symptoms such as behavioral disinhibition, apathy, loss of sympathy or empathy, and compulsive behaviour. FTLD-TDP is characterized by the presence of inclusions of hyperphosphorylated tau protein in neurons and glial cells, which is a pathological hallmark for the development and progression of neurodegenerative disorders. The most common neurodegenerative disorder that has been associated with the development of FTLD-TDP is sporadic amyotrophic lateral sclerosis (ALS). Found in about 5% of FTLD cases, FTLD-FUS present fused-in-sarcoma protein (FUS) positive cytoplasmic and intranuclear inclusions, which are commonly found in the frontal and temporal neocortex, and hippocampus [107,108,109]. The pathology of FTLD is heterogeneous and remains highly variable from patient-to-patient; however, the underlying neuropathological phenotypes that develop are mainly a result of CHMP2B alternative splicing.

In an exome mutation analysis of 146 patients with FTLD, PSP, or CBD, van der Zee et al. [50] found two mutations located within *CHMP2B* exon 5. The splice site mutation linked to chromosome 3 in *CHMP2B* produces a mutant protein referred to as CHMP2B^Intron5^, which lack the C-terminal 36 amino acids. Patients with this nonsense mutation developed early symptoms of FTLD at the age of 58. This C-terminal truncation of *CHMP2B* interferes with its charge distribution, which prevents the C-terminus from functioning as an autoinhibitory domain [50]. The reason for this is that the C-terminus of CHMP proteins undergo intramolecular interactions with their basic N-terminus to regulate the balance between their closed inactive soluble state versus open activated state [110,111,112]. As a result, this allows CHMP proteins to polymerize into heteromeric ESCRT-III complexes on the endosomal membrane. However, as a result of the C-terminal truncation, CHMP2B would be forced to remain in an active state, as the mutation prevents the inhibitory effect of the C-terminus. Thus, the binding of truncated proteins in ESCRT-III complexes persist and accumulate on the endosomal membrane. Furthermore, CHMP proteins typically interact with Vps4 AAA-ATPase on their C-terminus [112,113]. Thus, C-terminal truncation of CHMP2B would likely inhibit MVB formation due to failure to recruit VPS4, which is required to actively dissociate the ESCRT-III complex, which in turn would block invagination and ILV formation [50].

Mouse models carrying the CHMP2B^Intron5^ mutation form ubiquitin deposits and p62 aggregates in the brain because of impaired autophagy [114,115]. Recent work by Lu et al. revealed that syntaxin 13 is a genetic modifier of CHMP2B^Intron5^ in *Drosophila.* The group then showed that syntaxin 13 plays a role in the maturation of phagophores into closed autophagosomes [116]. Taken together, the CHMP2B splice mutant appears to dysregulate the early stages of the autophagy pathway and this likely contributes to the characteristic p62- and ubiquitin aggregates of FLTD. We previously discussed how reductions in CHMP3 levels resulted in TDP-43 aggregate formation. These authors also examined the well-studied CHMP2B splice mutant (CHMP2B^Intron5^) but did not see TDP-43 positive aggregates. The TDP-43 negative nature of the p62-positive aggregates with the CHMP2B splice mutant has been documented in patients [115]. The results suggest that CHMP3 and CHMP2B defects in autophagy result in unique neurodegenerative pathologies in FLTD; also, that their regulation of autophagy differs mechanistically. Thus, highlighting the importance of studying how the different ESCRT proteins contribute to the regulation of autophagy.

### 4.3. Autosomal Dominant Cataracts and CHMP4 Regulation of Autophagy-Mediated Micronuclei Clearance

In 2007, Shiels et al. [52] identified the *CHMP4B* gene as a key gene in the development of autosomal dominant cataracts. CHMP4B is one of several human paralogs of the yeast CHMP4 gene *Snf7*, which also includes hSnf7-1/CHMP4A, and hSnf7-3/CHMP4C [117]. The Snf7 family are coiled-coil-forming proteins that are involved in MVB formation, structure, and function, which are recruited from the cytoplasm to endosomal membranes where it oligomerizes into the ESCRT-III complex [117,118]. In yeast, mutations to Snf7p show defects in the late endosome to MVB transition, resulting in the inability to transport target proteins to the yeast vacuole [119].

To understand what role CHMP4 proteins may play in cataract development we need to first understand their cellular function. The human CHMP4 family members are late-acting components of the ESCRT pathway that share the ability to bind to ALG-2 interacting protein (ALIX, also known as BRO1) [120,121,122,123,124]. ALIX acts as an adaptor protein which functions in abscission by connecting CEP55 with the ESCRT-III complex [120,121]. Interestingly, CHMP4 proteins can recruit ALIX to membranes because the membrane bound Snf7p moves Bro1p/ALIX to the endosome to mediate MVB vesicle formation [122]. Moreover, ALIX point mutants that block CEP55 and CHMP4/ESCRT-III binding also inhibit abscission, indicating that its involvement is essential in ESCRT pathways [123]. A mutational analysis of the ALIX_Bro1_-CHMP4A interaction showed that single alanine substitutions of CHMP4A residues terminated ALIX_Bro1-V_ binding [122]. Therefore, the inability for ALIX to bind to CHMP4 will prevent the assembly of the ESCRT-III complex, which would overall disrupt the MVB biogenesis and ILVs formation, and consequently the sorting of ubiquitylated membrane proteins into vesicles [11,124].

**Figure 3:**
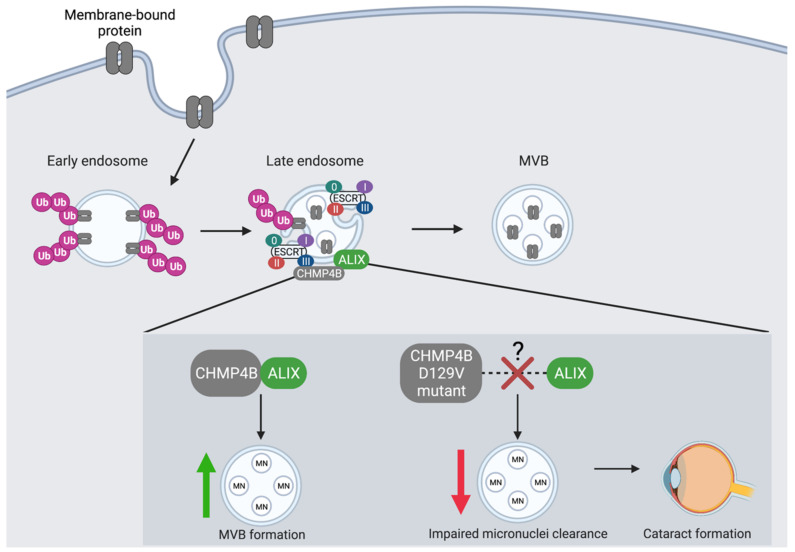
The co-localization of CHMP4B and ALIX is an essential regulatory event in the autophagic degradation of micronuclei. CHMP4 proteins are essential components of the MVB pathway where they interact with ESCRT-III to recruit the ALG-2 interacting protein (ALIX). CHMP4B shows the greatest binding affinity towards ALIX. CHMP4B plays an essential role in the recycling of micronuclei (MN), which is dependent upon its recruitment and interaction with ALIX at the endosome. CHMP4B localizes to micronuclei in the lens of the eye to facilitate autophagic degradation and prevent cataract formation. The D129V mutation in CHMP4B is linked to cataract formation likely as a result of a blocked interaction between CHMP4B and ALIX, which leads to decreased autophagic degradation of MVB cargo and accumulation of micronuclei.

Out of the different CHMP4 paralogs, CHMP4B shows the greatest binding affinity towards ALIX in human tissue [125] (Figure 3). CHMP4B plays an important role in both mitophagy [55] and the recycling of micronuclei and extranuclear chromatin through the autophagolysosome [53]. Interestingly, when CHMP4B was unable to localize with pAlixΔC-V5, the level of endogenous CHMP4B was low which resulted in the inability to induce vesicle formation [53]. Thus, co-localization of ALIX and CHMP4B may represent a key regulatory event in the autophagic degradation of MVB cargo [53]. However, while CHMP4B shows the greatest binding affinity towards ALIX, it does not suggest that autophagy is solely dependent upon this interaction. For instance, the binding of ALIX to CHMP4B may indirectly induce autophagy by aiding in the ILV formation [126,127]. Finally, depletion of CHMP4B using RNA interference has been shown to directly impair starvation-mediated degradation of autophagy receptors such as p62 coincident with loss of MVB structures [128], further implicating this CHMP4 paralog in autophagy degradation pathway.

The lens of the eye is composed of fibre cells, which to form a lens must become devoid of organelles, including the nucleus to ensure transparency [129,130]. Organelle clearance is believed to occur by autophagy in the lens [131]. However, removal of the nuclear DNA in the eye is also facilitated by DNase II-like acid DNase (DLAD, also known as DNASE2B) and in DLAD knock-out mice the failure to clear nuclear DNA results in cataract formation [132]. Previously, CHMP4B has been implicated in abscission of the intracellular bridge during cytokinesis and can be localized to DNA bridges and micronuclei during aberrant cell division [133,134]. Sagona et al. [53] also found that CHMP4B localizes to micronuclei in human tissue. These CHMP4B-positive micronuclei were also positive for Lamp1 and LC3, which are lysosomal and autophagic markers (respectively). Thus, these findings implicate CHMP4B in the autophagic degradation of micronuclei [53]. Furthermore, when Sagona and colleagues expressed the mutant form of CHMP4B (CHMP4B^D129V^) found associated with autosomal dominant posterior polar cataracts [52] (Table 1), interfered with the ability of CHMP4B to associate with micronuclei in Hela cells. Of interest, the D129V mutation in CHMP4B associated with the development of cataracts lies adjacent to the minimal interaction region of CHMP4B with ALIX (i.e., between amino acids 205 and 224) [120]. As such, it is tempting to speculate that the D129V mutation may also alter interaction between ALIX and CHMP4B, with implications for autophagy. Thus, it is possible that perturbed autophagy-mediated clearing of micronuclei occurs in lens fibre cells expressing CHMP4B^D129V^ and this contributes to cataracts in patients carrying this mutation (Figure 3). Given this link between CHMP4B and micronuclei surveillance and clearance, it will be important to evaluate the role of CHMP4B in other diseases states associated with micronuclei formation, including chromosomal instability seen in some cancers [135,136].

### 4.4. VPS4B Mis-Splicing Causes Dentin Dysplasia I

VPS4 plays an important role in promoting the association with and recycling of ESCRT-III proteins at endosomal compartments in the cell [137]. The AAA-type adenosine triphosphatase VPS4 constricts and cleaves ESCRT-III CHMP2A-CHMP3 filaments to set the stage for membrane fission [138]. In *Drosophila*, *Vps4* is required to promote autophagic flux at axons and helps prevent Wallerian degeneration, a controlled self-destruction process that injured axons undergo [139]. There are two mammalian paralogs of *Vps4*, *VPS4A* and *VPS4B*, and the role of *VPS4B* in regulating autophagy and Wallerian degeneration is conserved between flies and mammals [139]. This assumption is further supported by a mammalian ATPase-deficient dominant-negative mutant of VPS4B (referred to at the time as SKD1), which was shown to block autophagosome-lysosome fusion [140]. Reduction of ESCRT-III activity in a *Drosophila* model for Huntington’s disease, where a polyglutamine repeat protein is expressed in the photoreceptor neurons of the eye, was also show to exacerbate polyglutamine (polyQ)-induced neurotoxicity coincident with autophagosomes accumulation and impaired endo-lysosomal fusion [40] (Table 1). Therefore, there is a strong body of evidence that suggests an evolutionarily conserved role for ESCRT-III proteins and Vps4 in the regulation of autophagy and neurotoxicity.

The mis-splicing of VPS4B and consequent dysregulation in autophagy has also been linked to human disease. Dentin dysplasia I (DDI) is an autosomal-dominant disorder that results in rootless teeth and altered pulpal morphology in affected patients. It was found that patients with DDI harbored a mutation on chromosome 18 (18q21.2–q21.33) that created a novel donor splice site in intron 7 (IVS7 + 46C > G) of the *VPS4B* gene [51] (Table 1). This led to a 15 amino-acid insert that altered the ATP-binding cassette of VPS4B, reduced expression of its mRNA, and altered localization of the mutant protein. Knockdown of *Vps4* in zebrafish also resulted in a similar phenotype, where there was a reduction in tooth size and absence of teeth [51]. These results together indicate that *VPS4B* mis-splicing results in a loss-of-function scenario. However, when a heterozygous *Vps4b* KO was made in mice that that better modelled the heterogeneous genetic nature of DDI, there was no phenotype in tooth or bone development observed [141]. These results suggest that although the role of Vps4 in tooth development is conserved between fish and humans, its function in mouse tooth development is less prominent or lost.

A proposed mechanism for how mis-splicing of VPS4B manifests into DDI relates to osteogenic differentiation [142]. The mis-splicing mutation was examined in vitro using dental follicle cells that were isolated and cultured from a patient with DDI. The VPS4B IVS7 + 46C > G mutation altered cell proliferation and impaired osteogenesis, where key osteoblast-related genes were downregulated. Although the link between VPS4B and osteogenesis is still unclear, from a cellular perspective the dysregulation of autophagy in the absence of VPS4B is one potential mechanism. Supporting this hypothesis, other studies in human gingival fibroblasts (the cell model also used to characterize mis-spliced VPS4B) have demonstrated that autophagy is essential for driving osteogenic differentiation [143]. When cells were cultured in differentiation-stimulating media, there is a striking increase in autolysosomes. Since Vps4 is required for autophagosome-lysosome fusion, the loss of this function in the VPS4B splice mutant may help explain the pathology behind DDI. In conclusion, pathogenic splicing of VPS4B in DDI highlights the connected nature of ESCRT splicing and autophagy, which ultimately has important implications for human disease.

## 5. Concluding Remarks

**Figure 4:**
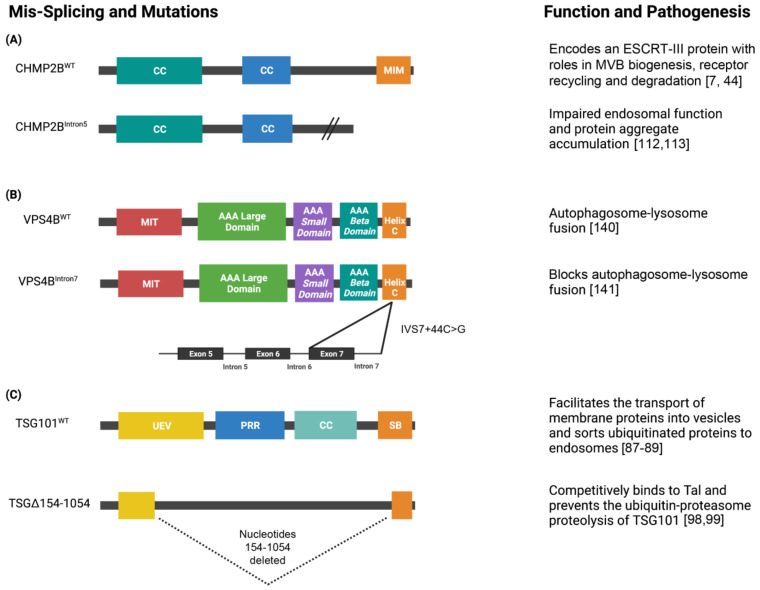
Schematics of the domain architecture of ESCRT proteins and their alternatively spliced isoforms. (**A**) CHMP2B is a 213 amino acid protein that contains two N-terminus coiled-coiled (CC) domains and a MIT-interacting motif (MIM) at its C-terminus. CHMP2B encodes an ESCRT-III protein that is essential for the biogenesis of multivesicular bodies (MVB’s) and sorting ubiquitinated proteins into intralumenal vesicles. The splice site mutation linked to chromosome 13 in CHMP2B produces the isoform CHMP2BIntron5. CHMP2BIntron5 forms a C-terminal truncated protein that lacks the MIM domain. The altered C-terminus impairs endosomal function and causes protein aggregate accumulation, which is a pathogenesis associated with FTLD-U, PSP, and CBD. (**B**) VPS4B is a 444 amino acid protein that contains a microtubule interacting and transport (MIT) domain that serves as an adapter for ESCRT-III proteins, large and small AAA ATPase domains, and a C-terminal helix domain. A mis-splicing event of VPS4B results in a novel donor splice site in intron 7 (IVS7 + 46C > G) which prevents autophagosome-lysosome fusion. (**C**) TSG101 is a 390 amino acid protein that contains a ubiquitin E2 variant (UEV) domain, proline-rich region (PRR), CC domain, and steadiness box (SB) at its C-terminus. TSG101 is a member of the ESCRT-I complex that facilitates the sorting of ubiquitinated proteins to endosomes and the formation of MVBs. TSG101 is often associated with aberrant transcripts that contain splicing abnormalities and the most common isoform is TSGΔ154-1054. TSGΔ154-1054 results from the deletion of nucleotides 154 to 1054 (dashed lines) and competitively binds to Tal which prevents the polyubiquitination and proteasomal degradation of TSG101. Created with BioRender.com.

Autophagy is a complex process that, despite tremendous effort, is still poorly understood in certain aspects, including its relationship with the ESCRT and pre-mRNA splicing pathways. It has been demonstrated that ESCRT is likely required for phagophore closure and likely autophagosome fusion, as defects in the ESCRT pathway can lead to failure of autophagosomes-lysosome fusion and result in autophagosome accumulation. Loss of ESCRT function and disrupted autophagy is associated with diseases such as autosomal dominant posterior polar cataract; a consequence of *CHMP4B* loss [52,53] (Figure 3). Alternative splicing also plays a role in the induction and maintenance of autophagy directly by altering splicing of autophagy-associated genes like *Beclin1* [65] (Table 1) or indirectly via the splicing of genes such as Heh1 that modulate subcellular recruitment of the yeast homolog of *CHMP7*, *Chmp7* [83] (Figure 2). Furthermore, alternative and/or mis-splicing of ESCRT genes that affect autophagy play roles in the pathogenesis of several diseases, including *TSG101* in cancer and *CHMP2B* and *VPS4B* mis-splicing in the pathogenesis of FLTD and dental dysplasia I (respectively) [101,141] (Figure 4). These findings also have implications for therapy. For example, the TSG101 TSGΔ154-1054 splice variant is aberrantly expressed in a range of human cancers and could be potentially targeted in these malignancies [90,92,93]. Given the knowledge gaps that remain, the coordinated regulation of alternative splicing, ESCRT, and autophagy represents an exciting avenue for future research with potential impact for disease linked to the dysregulation of autophagic pathways, including cancer, dementia and neurodegeneration (Table 1, Figure 4).

## Figures and Tables

**Table 1:** A summary of disease and pathogenesis that results from impaired or dysfunctional ESCRT complexes. Impaired function of the ESCRT complexes has been associated with cancer, neurodegenerative diseases, and cataract formation.

Complex	Subunit (Alternate Name)	Dysfunction/Disease	Pathogenesis
*Cancer* ESCRT-I	Tsg101 (Vps23, Bro1)	Tumorigenesis and metastasis	Functional inactivation of Tsg101: promote metastatic tumors in mice [47].
	TSG101 (Vps23, Bro1)	Cervical neoplasia	TSG101 splice variant transcription increased in cervical neoplasia [48].
	Tsg101 (Vps23, Bro1)	Mammary epithelial cancer	Overexpression of Tsg101 in mice increased activation of MAP kinases [49].
*Neurodegenerative diseases* ESCRT-III	CHMP2B (Vps24)	Frontotemporal lobar degeneration (FTLD-U), progressive supranuclear palsy (PSP), and corticobasal d	Impaired endosomal function and protein aggregate accumulation [50].
ESCRT-I/III	CHMP3 (Vps24), CHMP2B, Tsg101 (Vps23, Bro1)	FTLD-U and ALS	Depletion of CHMP3 prevents autolysosome formation and results in accumulation of Ub-protein aggregates containing TDP-43 [38].
ESCRT-I/III	Vps4	Huntington’s disease (HD)	Reduced ESCRT-III worsen polyglutamine-induced neurotoxicity in flies [40]
	VPS4B (SKD1)	Dentin Dysplasia type I (DDI)	Mis-splicing of VPS4B causes dentin dysplasia type I (DDI) [51]
ESCRT-III	CHMP4B (Snf7-2), CHMP2B	Neurodegeneration (FTLD-U and ALS)	Overabundance of autophagosomes. Mutants CHMP2B inhibits autoinhibitory domain, resulting in impaired endosomal function [50].
*Eye Disease* ESCRT-III	CHMP4B (Snf7-2)	Autosomal dominant posterior polar cataract	Absence of functional CHMP4B prevents micronuclei degradation [52,53].

**Table 2:** A summary of autophagy genes that are subject to alternative pre-mRNA splicing with consequences for autophagy.

Alternatively Spliced Genes	Autophagic Function/Outcome
*BCN1*	Splicing variants of *BCN1* converts it from an inducer of autophagy to a regulator of mitophagy [66].
*MAP1LC3B* (*LC3B*)	*MAP1LC3B* splice variant may prevent phagophore formation and incomplete closure of the phagophore [67].
*ATG5*	Cancer cells that express splice isoform of *ATG5* fails to induce autophagic response [66].
*ATG7*	Alternative splicing of *ATG7* results from mutations in the splicing factor U2AF35 and impairs autophagy initiation [66,68].
